# On the Successful Treatment of Consumptive Disorders, and Female Complaints Connected Therewith; on Scrofulous Diseases; and on the Management of Delicate Health by Diet and Regimen

**Published:** 1839-04

**Authors:** 


Art. XI.—*On the successful Treatment of Consumptive Disorders, and Female Complaints connected therewith; on Scrofulous Diseases; and on the Management of delicate Health by Diet and Regimen: with Cases.* By J. J. Furnivall, m.d., Physician to the General Infirmary at Hertford.—*London*, 1838. 8vo. pp. 320.

Whoever reads Dr. Furnivall's little volume attentively, as we have done, will, we are assured, feel with us that he has been very ill advised in publishing it at all; and yet far more so in prefixing to it the title transcribed above. Had we not been informed that Dr. F. is a gentleman above all suspicion, we should have been inclined to believe, on comparing the contents with the title-page, that the author was influenced by motives which an honorable physician would shrink from having attributed to him; so destitute of novelty are the former, so unhappily attractive is the latter. The greater part of the work is devoted to the hygienic treatment of "*delicate health*," in which term he includes tuberculous cachexia and incipient phthisis; but we are not aware that anything of importance is here noticed that the profession had not access to already, in the admirable treatise of Sir James Clark on Consumption, and in Dr. Combe's most valuable "Principles of Physiology applied to the Preservation of Health." Indeed, the volume might almost be regarded as an abridgment of these two works,—although that of Dr. Combe is only slightly referred to, and that of Sir James Clark not even mentioned,—interspersed with various theoretical notions of the author's own respecting the carbonization and decarbonization of the blood, debility of the organic nerves, animal electricity, &c. And, to say truth, bating these theories, the small volume contains much that is both true and valuable in regard to the preservation of health and the prophylaxis of disease, and will be useful to all those into whose hands it may fall who have not seen the works referred to, or others of the same class. Of the importance of such knowledge to the public generally, no one can be more convinced than ourselves; and, had not Dr. Furnivall provoked us by the unfortunate pretension of his title, we are not sure but we might have quietly recommended his little volume to the class of "general readers," as they are usually termed, without any depreciating criticism: for (to avail ourselves of the author's most quaint and *striking* metaphor) "if the anvil of the human frame, in some individuals, will not safely bear the strokes which the ordinary course of events must cast upon it, it is the duty of the medical practitioner to attempt to strengthen it [the anvil]; or else he ought to direct how such blows may be either safely borne, or altogether avoided."

After careful examination of the chapter on *treatment*—"*successful*" *treatment* it ought to have been, according to the title-page,—and perusal of all the cases, we are sorry to give it as our opinion that this volume contains not a particle of evidence of any greater success in the treatment of consumption than any practitioner, of even a few years' experience, could adduce; and that the medical means recommended are not only common, but scarcely even reaching the level of the ordinary treatment of the disease by scientific physicians in general. We may even add, with little fear of contradiction from practitioners of experience who are at once pathologists and logical reasoners, that not one of the cases detailed supplies any *proof* of the *cure* (properly so called) of consumption. In many of them there was no satisfactory evidence of the existence of tubercular phthisis at all; and, in the few which presented such evidence, the *very recent* date of the so-called cure vitiated every inference tending to support this view, by suggesting those temporary ameliorations and retardations of progress so conspicuous in a numerous class of these cases, under almost any treatment.

Dr. F.'s more general treatment seems to have consisted in the giving of a grain of calomel, combined sometimes with colocynth, sometimes with Dover's powder, once or twice and a bitter mixture, containing carbonate and sulphate of soda, twice or thrice a day; with occasional emetics, blisters, nitro-muriatic or iodine lotions, and other means in every-day use. We had marked for quotation some of Dr. F.'s cases, as specimens of his treatment, and as proofs of the justice of our remarks, but we are prevented from extracting them by want of room: had our object been to justify these remarks completely, we need only have transcribed the greater number of the cases in the volume.

